# News and Notes

**Published:** 1900-12

**Authors:** 


### *NEWS AND NOTES.*

---

Dr. Alfred Stengel has been appointed on the medical staff of the Pennsylvania Hospital in succession to the late Professor J. M. DaCosta.

---

It is stated that there are 30,000 lepers in the Philippines. It is believed that the disease was introduced in 1633 by a vessel from Japan, which brought 150 lepers to be placed under the care of the Catholic priests.

---

A banquet was recently held in Chicago in honor of the sixtieth birthday of the well-known surgeon and pathologist, Dr. Christian Fenger. This is a pleasing old-world custom which is becoming popular in this country.

---

In his address to the thirty-seventh annual convention of the American Veterinary Medical Association, held at Detroit, Mich., Dr. Leonard Pearson, of Philadelphia, urged that the efforts of all the members be concentrated during the ensuing year on the securing of commissions for veterinarians in the regular army.

---

Rush Medical College is to have a new building to cost $80,000, for which Dr. Nicholas Senn has just given $50,000. The building is to be called Senn Hall. Primarily, the new building will be for clinical purposes. In addition to complete arrangements for clinical work, there will be class-rooms and laboratories.

---

We are pleased to note that both Messrs. Breitenbach and Welles, of M. J. Breitenbach Co., New York, manufacturers of

Gude's Pepto-Mangan, escaped injury in the recent explosion in the Tarrent building. They write us the market is supplied with Gude's Pepto-Mangan, and with true American spirit will be on their feet again in ten days.

---

A HANDSOME and commodious infirmary has recently been completed in Little Rock, Ark., by the Sisters of Charity of that place. The dedicatory address was, by invitation, delivered by our esteemed colleague of the tripod, Dr. James T. Jelks, editor of the *Hot Springs Medical Journal*, an organ which is an excellent representative of its section.

---

MISSISSIPPI VALLEY MEDICAL ASSOCIATION.—Next place of meeting, Put-in-Bay, Ohio, September 10, 11, and 12, 1901. President, A. H. Cordier, of Kansas City, Mo.; first vice-president, C. F. MaGahan, of Aiken, S. C.; second vice-president, Charles L. Minor, of Asheville, N. C.; secretary, Henry E. Tulley, of Louisville, Ky.; treasurer, Dudley S. Reynolds, of Louisville, Ky.; Chairman of the Committee of Arrangements, J. C. Culbertson, of Cincinnati, O.

---

WE learn from the *Lancet* that the German Emperor has recently conferred upon Sir William MacCormac the "Kaiser Wilhelm" memorial medal in recognition of his services during the Franco-German war of 1870–71, and Her Majesty, the Queen, has been graciously pleased to grant her permission to him to accept and wear the medal. The medal was not long since instituted by the present emperor in memory of his grandfather, "Der Grosse Kaiser."

---

EDWARD R. SQUIBB, M.D., Jefferson Medical College, 1845, died at his home in Brooklyn, October 26, aged 81 years. He was born in Wilmington, Del., and was in charge of the medical station at the Brooklyn navy yard when the Civil War broke out. On his resignation from the navy he started a private laboratory in Brooklyn. He retired from business about fifteen years ago. He was a member of The American Medical Association, New York State Medical Association, etc. Dr. Squibb was an authority on pharmaceutical subjects.

---

THE State Board of Medical Examiners of New Jersey, on July 5th, 1900, adopted the following resolution: "*Resolved*, That this Board will indorse the license of any State Board of Medical Examiners in the United States, in lieu of an examination; provided, first, that the candidates for indorsement shall present satisfactory evidence of having the academic and medical education required by this Board, and that the license presented for indorsement shall have been issued after a State examination of the same grade and kind as that required by this Board."

---

DR. R. BEVERLY COLE, coroner of San Francisco, was entertained at a banquet in his honor recently in the University Club by the faculty of the Medical Department of the University of California. Dr. Cole was for many years professor in the medical department, and the faculty has decided to lighten his work, owing to ill-health. He has been appointed Emeritus Professor of Obstetrics and Gynecolgy. After the banquet Dr. Cole was presented with a handsome testimonial in recognition of his services. It consisted of a beautifully engraved silver plate.

---

MANY members of the medical profession are interested in the study of American ethnology and archeology, and not a few have valuable collections of Indian relics and skeletons from Indian graves. Those not directly interested in this study are so circumstanced as to be aware of the hobbies of their neighbors and could doubtless furnish the address of collectors. Dr. A. L. Benedict, of Buffalo, N. Y., having been appointed superintendent of Ethnology and Archeology at the Pan-American Exposition to be held at Buffalo, N. Y., during 1901, desires information and the loan of collections for the use of this department of the exposition. Exhibits which represent study in some special line of American ethnology and archeology will be particularly suitable.

---

THOUGH Time may dig the grave of creeds
  And dogmas wither in the sod,
My soul will keep the thought it needs—
  Its swerveless faith in God.

No matter how the world began
  Nor where the march of science goes,
My trust in something more than man
  Shall help me bear life's woes.

Let progress take the props away
  And mouldering superstitions fall;
Still God retains his regal sway—
  The maker of the All.

Why cavil over that or this?
  One thought is vast enough for me—
The great Creator was and is
  And ever more shall be.

---

THE next meeting of the Pan-American Medical Congress will be held in the City of Havana, Cuba, on December 26th, 27th, 28th, and 29th. The congress is organized in twenty-two sections, as follows: Medicine, general surgery, military medicine and surgery, obstetrics, gynecology and abdominal surgery, therapeutics, anatomy, physiology, pediatrics, pathology, ophthalmology, laryngology and rhinology, otology, dermatology and syphilography, general hygiene and demography, maritime hygiene and quarantine, orthopedic surgery, mental and nervous diseases, dental and buccal surgery, medical pedagogy, medical jurisprudence, and railway surgery.

---

WE learn from the *Medical Record* that the University of Dallas has recently received its charter from the State of Texas, and the first Board of Directors has been elected. At present only the School of Medicine will be inaugurated. The faculty consists of the following physicians: Drs. J. E. Gilchrist of Gainesville; V. B. Armstrong of Dallas; B. E. Hadra (President Texas Medical Association) of Waco; S. E. Milliken of Dallas; Joe Becton of Greenville; L. Ashton of Dallas; C. M. Rosser of Dallas; A. F. Beddo of Dallas; J. B. Titterington of Dallas, dean of the faculty.

---

THE Tri-State Medical Society of Alabama, Georgia and Tennessee held its twelfth annual meeting in Chattanooga, October 11th, 12th and 13th. The President's address, by Dr. Rufus R. Kime, Atlanta, Ga., was entitled "The Relation of the Profession to the Public." He dwelt on the duty of the profession to protect the public against their own folly in the use of dangerous drugs, coal-tar derivatives, opium, alcohol, etc., and suggested that a committee be appointed for the study of social problems affecting the development of the race. The following officers were elected: Dr. Matthew C. McGannon, Nashville, president; Dr. Walter G. Bogart, Chattanooga, Dr. S. Harris, Union Springs, Ala., and Dr. Michael Hoke, Atlanta, vice-presidents; Dr. Frank Trester Smith, Chattanooga, secretary, and Dr. George R. West, Chattanooga, treasurer. Nashville was selected as the next place of meeting.

---

A PARAGRAPH in the *Journal of the American Medical Association* from Galveston says: The heroism of Dr. I. M. Cline, United

States Weather Observer at Galveston, a member of the Texas State Medical Association, stands out brightly among the many heroic deeds recorded there. At noon he sent out warnings that a disastrous storm was imminent. Three hours later, realizing that an unparalleled storm was approaching, he went through the southern part of the city imploring the people to fly to the center of the island. As the telephone and telegraph systems were then wrecked, he made his way to the telephone station at the bridge west of the city and sent an appeal for aid, just before the cable parted. On his return, after this duty had been performed, he found his house destroyed and his wife and one child dead.

---

THE committee on the Senn medal beg leave to call attention to the following conditions governing the competition for this medal for 1901:

1. A gold medal of suitable design is to be conferred upon the member of the American Medical Association who shall present the best essay upon some surgical subject.

2. This medal will be known as the Nicholas Senn Prize Medal.

3. The award shall be made under the following conditions: *a.* The name of the author of each competing essay shall be enclosed in a sealed envelope bearing a suitable motto or device, the essay itself bearing the same motto or device. The title of the successful essay and the motto or device is to be read at the meeting at which the award is made, and the corresponding envelope is to be then and there opened and the name of the successful author announced. *b.* All successful essays become the property of the Association. *c.* The medal shall be conferred and honorable mention made of the two other essays considered worthy of this distinction, at a general meeting of the Association. *d.* The competition is to be confined to those who at the time of entering the competition, as well as at the time of conferring the medal, shall be members of the American Medical Association. *e.* The competition for the medal will be closed three months before the next annual meeting of the American Medical Association, and no essays will be received after March 1, 1901.

Communications may be addressed to any member of the committee, consisting of the following: Dr. Maurice H. Richardson, Boston, Mass.; Dr. Frederick Holme Wiggin, New York, N. Y.; Dr. Clayton Parkhill, Denver, Col.

---

THE committee on the award of the Association medal desires to call attention to the following: The Amercian Medical Association offers annually a gold medal valued at $100.00 for the best essay on any subject relating to medicine or surgery. At the last meeting of the Association it was decided that hereafter the recipient of the prize should be given the option of the gold medal or a bronze replica of the medal and the balance of the appropriation in money.

The competing essays must be typewritten or printed, and bear no mark revealing their authorship; but instead of the name of the author, there must appear on each essay a motto, and accompanying each essay a sealed envelope containing the name of the author and bearing on its outer surface the motto of identification· No envelope is to be opened by the committee until a decision has been reached as to the most deserving essay, and the other essays have been returned to their respective owners. The committee has authority to reject and return all essays in case none have been found worthy of the Association medal.

The committee suggests as one of the important topics of the day that of Tropical Diseases, but, while suggesting this, does not wish to dictate in the slightest degree that this branch of medicine must be the subject discussed.

Competing essays must be in the hands of the committee not later than March 1, 1901. For further information address any member of the committee, which consists of the following: Dr. William Osler, Baltimore, Md.; Dr. C. W. Richardson, Washington, D. C.; Dr. Rudolph Matas, New Orleans, La.

